# Metal Substrate-Induced Line Width Compression in the Magnetic Dipole Resonance of a Silicon Nanosphere Illuminated by a Focused Azimuthally Polarized Beam

**DOI:** 10.1186/s11671-018-2796-7

**Published:** 2018-12-05

**Authors:** Fu Deng, Hongfeng Liu, Sheng Lan

**Affiliations:** Guangdong Provincial Key Laboratory of Nanophotonic Functional Materials and Devices, School of Information and Optoelectronic Science and Engineering, South China Normal University, Guangzhou, 510006 China

**Keywords:** Silicon nanoparticle, Magnetic dipole resonance, Azimuthally polarized beam, Metal substrate, Image dipole

## Abstract

We investigate the modification of the magnetic dipole resonance of a silicon nanosphere, which is illuminated by a focused azimuthally polarized beam, induced by a metal substrate. It is found that the magnetic dipole of the silicon nanosphere excited by the focused azimuthally polarized beam and its image dipole induced by the metal substrate are out of phase. The interference of these two anti-parallel dipoles leads to a dramatic line width compression in the magnetic dipole resonance, manifested directly in the scattering spectrum of the silicon nanosphere. The quality factor of the modified magnetic dipole resonance is enhanced by a factor of ∼ 2.5 from ∼ 14.62 to ∼ 37.25 as compared with that of the silicon nanosphere in free space. Our findings are helpful for understanding the mode hybridization in the silicon nanosphere placed on a metal substrate and illuminated by a focused azimuthally polarized beam and useful for designing photonic functional devices such as nanoscale sensors and color displayers.

## Background

Dielectric nanoparticles with large refractive indices and diameters ranging from 100 to 250 nm, which support distinct Mie resonances in the visible to near-infrared spectral range, have become the focus of many studies in recent years because they are considered as the promising building blocks for metamaterials working at optical frequencies [1–7]. The coexistence of magnetic dipole (MD) and electric dipole (ED) as well as their coherent interaction in such nanoparticles leads to many intriguing phenomena such as the enhanced and suppressed forward and backward scattering at specific wavelengths (e.g., the wavelengths satisfying the first and second Kerker’s conditions) [8–12]. Moreover, the interference between the electric and magnetic multipole modes can result in extraordinary directional scattering into different directions [13–15].

The electric and magnetic resonances excited in dielectric nanoparticles with large refractive indices can be manipulated by using various methods [16–31]. This unique feature offers us the opportunity to modify the linear and nonlinear optical properties of single nanoparticles and metamaterials composed of such nanoparticles. For example, the electric and magnetic resonances excited in a nanoparticle can be easily modified by changing its size or shape [16–25]. In addition, it has been shown that the substrate used to support a nanoparticle can also be employed to manipulate the optical responses of the nanoparticle. Particularly, the particle-film hybrid systems in which a dielectric nanoparticle is placed on a metal substrate have attracted great interest due to the formation of new resonant modes originating from the coherent interaction between the multipole modes of the dielectric nanoparticle and their mirror images induced by the metal substrate [26–32]. Under the excitation of a linear-polarized light, the interference of the ED of a Si nanosphere (NS) and its mirror image induced by a Au film leads to the formation of a MD located at the contact point between the Si NS and the Au film, where the magnetic field is significantly enhanced [26–29]. In case of oblique incidence, the line width of the mirror-image-induced MD in the Si NS can be controlled by varying the polarization of the incident beam [30].

Apart from substrate, structured light such as cylindrical vector beam acts as a powerful tool for manipulating the optical responses of dielectric nanoparticles [33–42]. For example, the selective excitation of the ED or MD resonance of a nanoparticle by using radially polarized or azimuthally polarized (AP) beams have been studied [35–42]. When a nanoparticle is placed at the focal point of an AP beam, only the magnetic modes of the nanoparticle are excited, and all the electric ones are suppressed because of the zero electric field along the beam axis [38–42]. For this reason, the magnetic resonances of the dielectric nanoparticle can be selectively excited, and the ideal anapole modes of magnetic type can be also activated by using 4 *π*-illumination with two AP beams [42]. Moreover, the MD modes of dielectric nanoparticles excited by a focused AP beam provide a perfect platform for tailoring the MD transition [43, 44].

So far, the studies on the scattering properties of Si NSs illuminated by using focused AP beam are suspended in the air or placed on SiO_2_ substrate [38–42]. The line widths of the MD resonances of such Si NSs still not satisfied for the practical applications where MD resonances with narrow line widths or large quality factors are highly desirable. For instance, a small increase in the quality factor of the MD resonance may lead to a significant enhancement in the two- and three-photon-induced absorption of Si nanoparticles, lighting up Si nanoparticles with femtosecond laser pulses [45]. Here, we investigate the scattering properties of a Si NS placed on a metal substrate and illuminated by a focused AP beam. Due to the rotational symmetry of the AP beam and the Si NS, only the magnetic multipoles of the Si NS are excited. It is found that the MD and its image induced by the metal substrate are out of phase, and the coherent interaction of them leads to a dramatic narrowing of the MD resonance (∼ 20 nm) as compared with that of the Si NS suspended in air (∼ 53 nm). Accordingly, the quality factor of the MD resonance is enhanced by a factor of ∼ 2.5 from ∼ 14.62 to ∼ 37.25. The sharp MD resonance achieved in the Si NS by using the combination of a metal substrate and a focused AP beam may find potential applications in nanoscale photonic devices such as sensors and color displayers.

## Numerical Methods

The scattering spectra of the Si NSs studied in this work were calculated by using the finite-difference time-domain (FDTD) method [46]. In the numerical calculations, the electric field of the AP beam at the focal plane was firstly calculated by the *k*-space beam profile definition [47] and then used for the FDTD simulation. The radius of the Si NS was fixed at *R* = 100 nm, and the metal substrate was chosen to be a perfect electric conductor (PEC) in the “Results and Discussion” and “Image Theory of the Out of Plane MD” sections and Au in the “Practical Applications” section. The optical constants of Si and Au were taken from Palik and Ghosh [48] and from Johnson and Christy [49], respectively. The surrounding medium of the Si NS was assumed to be air with a refractive index of *n* = 1.0. A mesh size of 3 nm was used in the illuminated region, and perfectly matched layers were employed at the boundary to terminate the finite simulation region.

## Results and Discussion

**Fig. 1:**
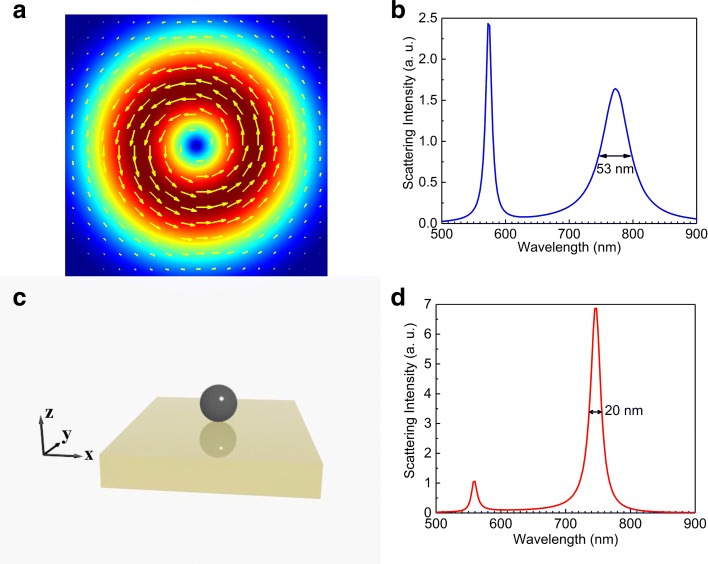
**a** The electric field distribution of a focused AP beam at the focal point. **b** The scattering spectrum of the Si NS suspended in air. The line width of the MD resonance is 53 nm. **c** The Si NS with *R* = 100 nm placed on a metal substrate. **d** The scattering spectrum of the Si NS placed on a PEC substrate

In Fig. 1a, we show the electric field distribution calculated for a focused AP beam at the focal plane. It is noticed that the AP beam possesses a rotational symmetry with zero electric field at the focal point (or along the axis). The electric field of the AP beam matches well with that of the Si NS at the MD resonance. In Fig. 1b, d, we present the scattering spectra calculated for the Si NS suspended in air and that placed on a PEC substrate, respectively. In both cases, it is remarkable that only the MD and magnetic quadrupole (MQ) resonances are excited, and all the electric resonances are suppressed, which is in agreement with the previous findings [38–42]. This behavior can be explained explicitly by using the multipole theory for tightly focused AP beam [42, 50]. If we compare the scattering spectra shown in Fig. 1b, d, it is found that the introduction of the PEC substrate leads to a dramatic narrowing of the MD resonance (from ∼ 53 to ∼ 20 nm). As a result, the quality factor of the MD resonance is enhanced by a factor of ∼ 2.5 (from ∼ 14.62 to ∼ 37.25).

In order to gain a deep insight into the modification of the scattering spectrum induced by the metal substrate, we decomposed the total scattering of the Si NSs into the contributions of various magnetic modes in a Cartesian coordinate [16, 25]. The polarization induced by the incident light is **P**=*ε*_0_(*ε*_*p*_−*ε*_*d*_)**E**, where *ε*_0_,*ε*_*p*_, and *ε*_*d*_ are the vacuum dielectric constant, relative dielectric permittivity of the Si NS, and relative dielectric permittivity of the surrounding medium, respectively, and **E** is the total electric field inside the Si NS. The time dependence of the incident light is assumed as exp(−*iωt*) with *ω* the angular frequency. The multipoles are defined in a Cartesian coordinate with the origin located at the center of the Si NS, and multipole moments can be obtained by the integration of the induced polarization currents over the volume of the Si NS. Thus, the MD moment and MQ tensor of the Si NS are described as:

1 $$\begin{array}{@{}rcl@{}} {\mathbf{M}} = - \frac{{i\omega}}{2}\int_{V} {{\varepsilon_{0}}\left({{\varepsilon_{p}} - {\varepsilon_{d}}} \right)\left[ {{\mathbf{r}}^{\prime} \times {\mathbf{\mathrm{E}}}\left({{\mathbf{r}}^{\prime}} \right)} \right]} d{\mathbf{r}}^{\prime}, \end{array} $$

2 $$\begin{array}{@{}rcl@{}} \widehat {\text{MQ}} = \frac{\omega}{{3i}}\int_{V} {\left\{{\left[{{\mathbf{r}}^{\prime} \times {\mathbf{P}}\left({{\mathbf{r}}^{\prime}}\right)}\right]{\mathbf{r}}^{\prime}}\right.\left.{+ {\mathbf{r}}^{\prime}\left[{{\mathbf{r}}^{\prime} \times {\mathbf{P}}\left({{\mathbf{r}}^{\prime}}\right)}\right]}\right\}} d{\mathbf{r}}^{\prime}, \end{array} $$

where *V* is the volume of the Si NS, and **r**^′^ is the radius vector of a volume element inside the Si NS.

The scattering cross sections of the MD and MQ can be expressed as follows [25]:

3 $$\begin{array}{@{}rcl@{}} {\sigma_{M}} = \frac{{k_{0}^{4}{\varepsilon_{d}}{\mu_{0}}}}{{6\pi {\varepsilon_{0}}{{\left|{{{\mathbf{{E}}}_{{\mathbf{inc}}}}} \right|}^{2}}}}{\left|{\mathbf{M}}\right|^{2}}, \end{array} $$

4 $$\begin{array}{@{}rcl@{}} {\sigma_{\text{MQ}}} = \frac{{k_{0}^{6}\varepsilon_{d}^{2}{\mu_{0}}}}{{80\pi {\varepsilon_{0}}{{\left| {{{\mathbf{{E}}}_{{\mathbf{inc}}}}} \right|}^{2}}}}{\left| {{\text{MQ}_{\alpha \beta }}} \right|^{2}}, \end{array} $$

where *μ*_0_ is the vacuum permeability, and the indexes *α*,*β*=*x*,*y*,*z*.

**Fig. 2:**
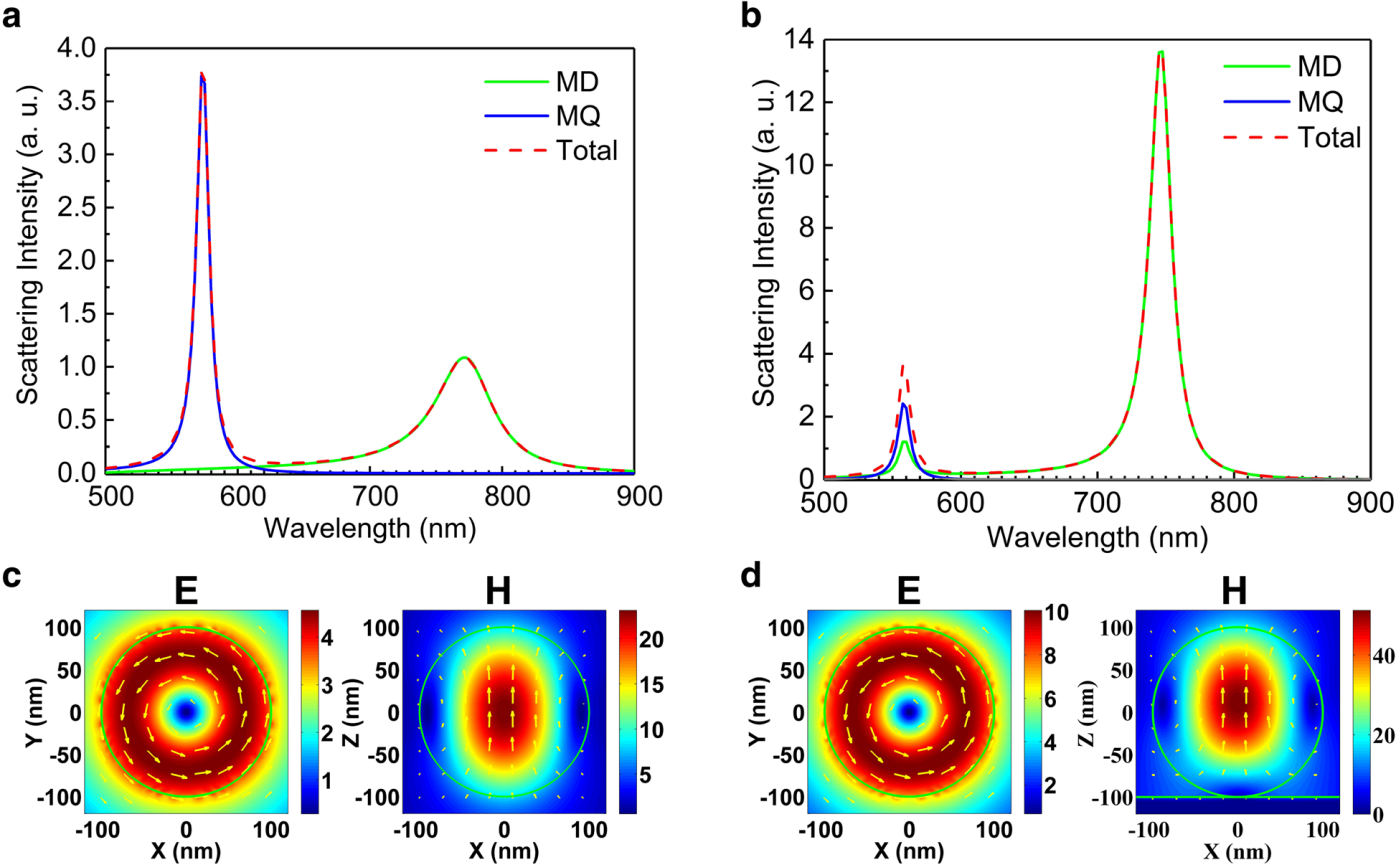
Multipole decomposition of the total scattering of the Si NS with *R* = 100 nm suspended in air (**a**), placed on a PEC substrate (**b**), and illuminated by a focused AP beam. The corresponding electric and magnetic field distributions calculated at the MD resonances [775 nm in **a** and 745 nm in **b**] are presented in **c** and **d**, respectively

In Fig. 2, we compare the multipole decompositions performed for the Si NS without and with the PEC substrate. In both cases, it can be seen that the total scattering is composed of only the contributions from MD and MQ modes. In addition, it is found that the narrowing of line width appears only in the MD resonance. In Fig. 2c, d, we present the electric and magnetic field distributions calculated for the two Si NSs at the MD resonances. It is noticed that the MD excited in the Si NS oriented in the +*z* direction in both cases. In addition, a significant enhancement is observed in the electric and magnetic fields of the Si NS in the presence of the PEC substrate.

## Image Theory of the Out of Plane MD

The narrowing of the MD line width can be understood by using the image theory and the approach based on Green’s function [27, 30]. We consider a MD located at the position **r**_**0**_=[*x*_0_,*y*_0_,*z*_0_] and the interface between air and the PEC substrate in the *x*−*y* plane with *z*=0. The magnetic moment is given by:

5 $$\begin{array}{@{}rcl@{}} {\mathbf{m}} = {\widehat \alpha_{m}}{{\mathbf{H}}_{\mathbf{0}}}, \end{array} $$

where ${\widehat \alpha _{m}} = \frac {{{\alpha _{h}}}}{{1 - {\alpha _{h}}{G_{M}}}}$ is the polarizability determined by the *z* component of the dyadic Green’s functions for the PEC substrate ${G_{M}} = \frac {{2i{k_{0}}{z_{0}} - 1}}{{16\pi z_{0}^{3}}}$ [30], and the polarizability of the Si NS is ${\alpha _{h}} = 6i\pi {b_{1}}/k_{0}^{3}$, *b*_1_ and *k*_0_ are the Mie coefficient and vacuum wavenumber, respectively.

The magnetic field at the center of the MD is given by: **H**_**0**_=[0,0, cos(*k*_0_*z*_0_)].

The extinction cross section of the MD can be written as [27]:

6 $$\begin{array}{@{}rcl@{}} {\sigma_{m}} = \frac{\omega}{{2{P_{\text{in}}}}}{{\text{Im}}}\left({{\mathbf{mH}}_{0}^{*}} \right), \end{array} $$

where *P*_*in*_ denotes the power of the incident light.

**Fig. 3:**
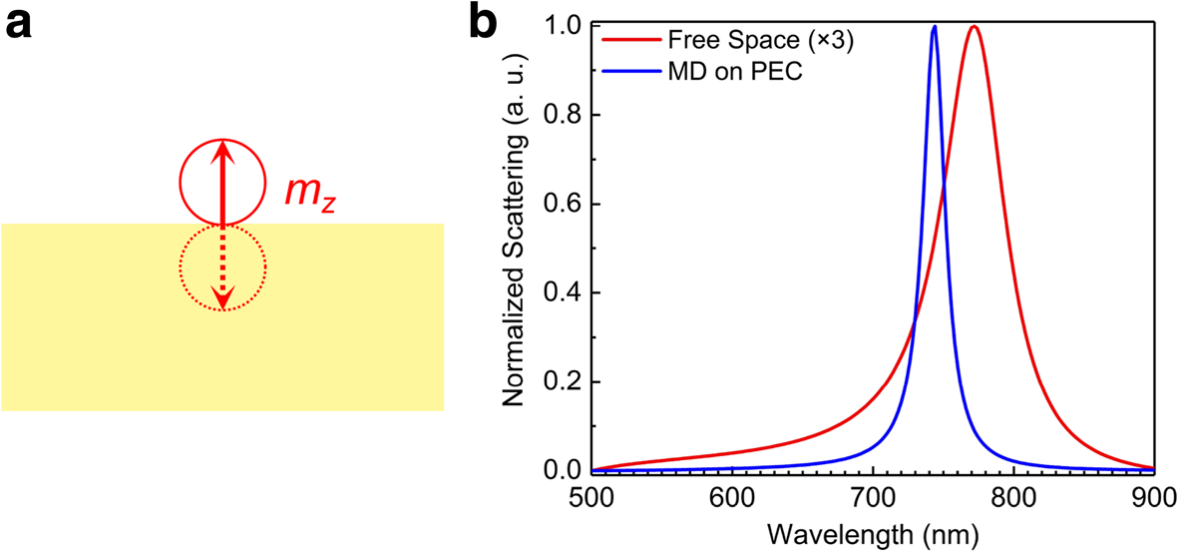
**a** Schematically showing the *z* component of MD excited in the Si NS and the mirror image induced by the metal substrate and their phase relationship. **b** Normalized scattering spectra calculated for the Si NS with *R* = 100 nm suspended in air and placed on a PEC substrate by using the dyadic Green’s function method

Due to the rotational symmetry of the AP beam and the Si NS, a MD oriented in the +*z* direction is excited in the Si NS. Meanwhile, a mirror image oriented in the −*z* direction is induced by the PEC substrate, as schematically shown in Fig. 3a. In this case, the displacement current is inverted in the mirror image, implying that the MD and its mirror image are out of phase. Thus, the coherent interaction of these two anti-phase MDs dramatically reduces the radiative loss, leading to the narrowing of the MD resonance in the scattering spectrum of the Si NS [30]. In Fig. 3b, we compare the MD resonances calculated by using the dyadic Green’s function method without and with the PEC substrate. In addition to the narrowing of the line width, a blue shift of the resonant wavelength as well as an increase in the scattering intensity (by a factor of ∼ 3.0) is also observed in the Si NS placed on the PEC substrate. The theoretical prediction shown in Fig. 3b is in good agreement with the numerical result shown in Fig. 1d. Therefore, the line width compression in the magnetic dipole resonance of the Si NS placed on the metal substrate illuminated by an AP beam can be perfectly explained by the image theory and the approach based on Green’s function.

## Practical Applications

In the above studies, it has been demonstrated theoretically and numerically that a sharp MD resonance can be created in the scattering spectrum of a Si NS by using the combination of a metal substrate and an AP beam. As some examples, we will show in the following numerical simulation the possible applications of the sharp MD resonance in nanoscale sensing and color display. For practical applications, the metal substrate is chosen to be a 50- nm-thick Au film, which has been used in our previous study [28]. The physical mechanism for the line width compression of the magnetic dipole resonance is the coherent interaction of the magnetic dipole and its mirror image induced by the metal substrate. Therefore, the material of the substrate should be metal but it is not limited to Au film.

### Sensor

**Fig. 4:**
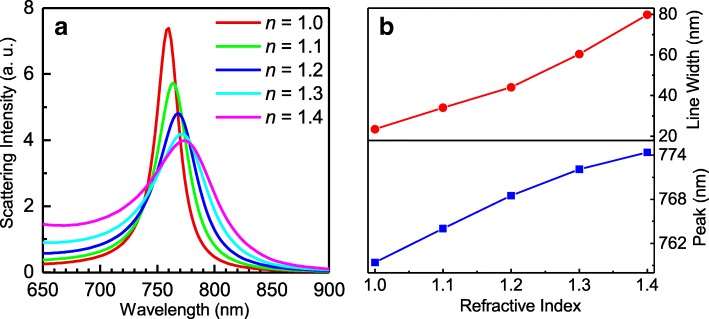
**a** Evolution of the scattering spectrum of the Si NS placed on the 50- nm-thick Au substrate with increasing refractive index of the surrounding medium. **b** Dependence of the line width (upper part) and the peak wavelength (lower part) of the MD resonance on the refractive index of the surrounding medium

Previously, it has been demonstrated that intensity shift sensors based on Si NS dimers possess much higher sensitivity than wavelength shift sensors based on plasmonic nanoparticles/nanostructures [51]. In addition, the sensitivity of the Si NS placed on a metal substrate and excited by linearly polarized light was also experimentally studied in our previous work [28]. In our case, the scattering spectrum dominated by a sharp MD resonance with a narrow line width is quite suitable for sensing applications, as demonstrated in the following. The sharp MD resonance is expected to be sensitive to the surrounding environment of the Si NS because it is created by the MD of the Si NS and its mirror image. Any change in the surrounding environment will lead to the modification in the MD resonance. In order to examine the sensitivity of the MD resonance, we calculated the evolution of the scattering spectrum of the Si NS with increasing refractive index of the surrounding environment, as shown in Fig. 4a. It is found that a slight change in the surrounding environment of the Si NS will result in a significant broadening and obvious red shift of the MD resonance, which can be seen clearly in Fig. 4b. Since the refractive index sensor proposed here detects the refractive index change in the surrounding environment, the ligands on the surface of the nanoparticle induced in the syhthesis process does not affect the detection function of the sensor. This feature is quite useful for sensing small specimens attached on the Si NS.

### Color Display

**Fig. 5:**
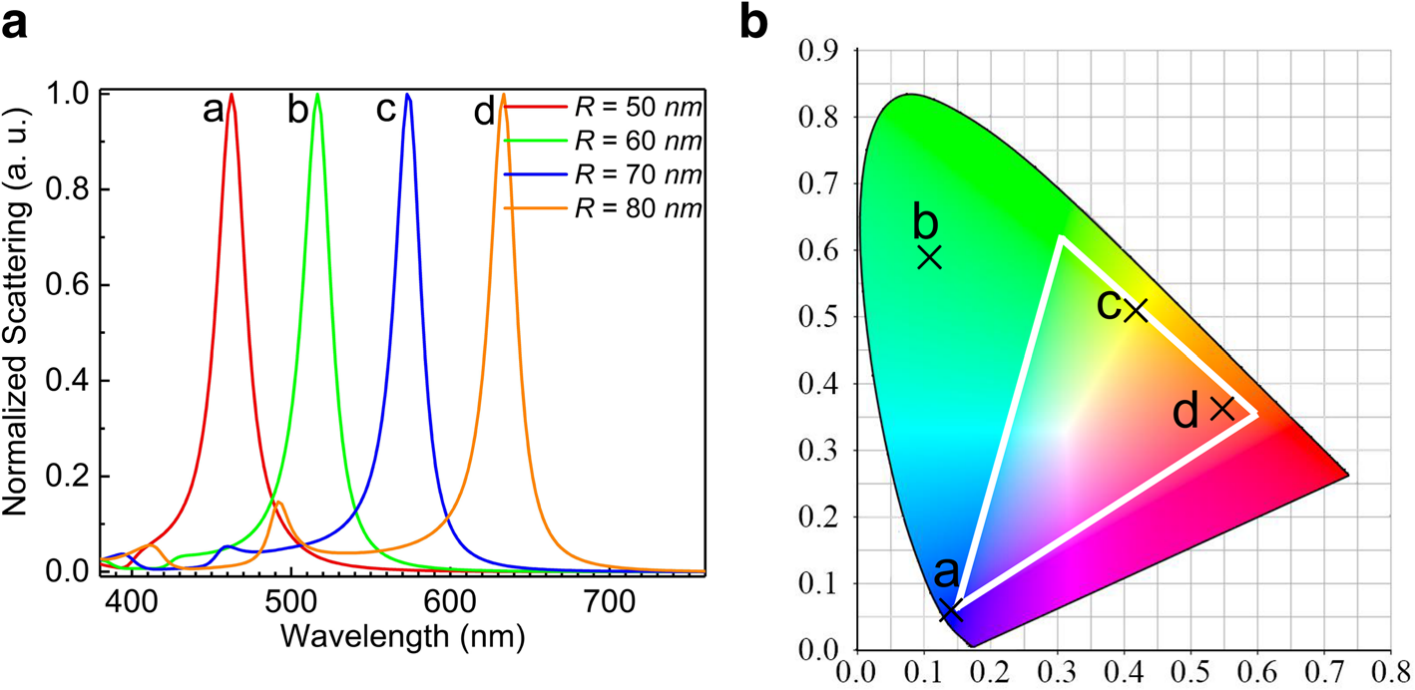
**a** Normalized scattering spectra calculated for Si NSs with different radii placed on a 50- nm-thick Au film. **b** Color indices derived from the scattering spectra shown in **a**

Recently, it has been successfully demonstrated that color control can be realized by using dielectric nanoparticles with large refractive indices, which supporting Mie resonances, instead of lossy plasmonic nanoparticles/nanostructures [52–55]. However, the ED and MD resonances of a Si NS are simultaneously excited in both bright- and dark-field illuminations, leading to broadband scattering light [52]. In a recent study, we have proposed a novel strategy for realizing color-tuning display with high spatial resolution and good chromaticity by using an evanescent wave to selectively excite the ED or MD resonance in the scattering spectrum of a Si nanoparticle [55]. Similarly, the sharp MD resonance found in this work is expected to be useful for color display because of the narrow line width and the enhanced scattering intensity. A significantly improved chromaticity is anticipated if the sharp MD resonance is used in color display. Moreover, high spatial resolution can be achieved because the enhanced scattering intensity enables the use of smaller pixels for color display. In Fig. 5a, we show the color tuning simply realized by varying the radius of the Si NS. It can be seen that a MD resonance with narrow line width can be achieved in all cases. In Fig. 5b, we present the color indices calculated for all the Si NSs with different radii. It can be seen that the color indices are distributed around the RGB triangle, implying the good chormaticity of the structral color produced by the Si NSs placed on the Au film. For practical application of color display, an array of Si nanoparticles instead of a single Si nanoparticle must be used. In this case, the line width of a single Si nanoparticle remains to be narrow provided that the coupling between the neighboring nanoparticles is negligible. According to the previous study [56], the coupling between Si nanoparticles in an array can be neglected when the separation between the neighboring nanoparticles is larger than 400 nm which is easily satisfied in practical fabrication.

## Conclusion

In summary, we have investigated theoretically and numerically the dramatic narrowing of the MD resonance of a Si NS, which is illuminated by using a focused AP beam, when placing it on a metal substrate. Owing to the rotational symmetry of the AP beam and the Si NS, only the multipoles of magnetic type are excited. It is found that the interference of the MD and its mirror image induced by the metal substrate is responsible for the dramatic narrowing of the line width from ∼ 53 to ∼ 20 nm. It is shown by numerical simulation that the sharp MD resonance in the scattering spectrum of the Si NS may find applications in nanoscale sensing with high sensitivity and color display with improved chromaticity and spatial resolution.

## Abbreviations

AP: Azimuthally polarized
Au: Gold
ED: Electric dipole
FDTD: Finite-difference time-domain
MD: Magnetic dipole
MQ: Magnetic quadrupole
NS: Nanosphere
PEC: Perfect electric conductor
Si: Silicon
